# Supplementary material for: Physical and functional interaction of Lrrc56 and Odad3 controls deployment of axonemal dyneins in vertebrate multiciliated cells
Source: bioRxiv. 2025 Jul 3:2025.07.02.662827. Preprint. [Version 1] doi: 10.1101/2025.07.02.662827 (PMC12236596; doi:10.1101/2025.07.02.662827)
Supplement: 2 [file NIHPP2025.07.02.662827v1-supplement-2.pdf]

bioRxiv preprint doi: https://doi.org/10.1101/2025.07.02.662827; this version posted July 5, 2025. The copyright holder for this preprint (which was not certified by peer review) is the author/funder, who has granted bioRxiv a license to display the preprint in perpetuity. It is made available under aCC-BY 4.0 International license.

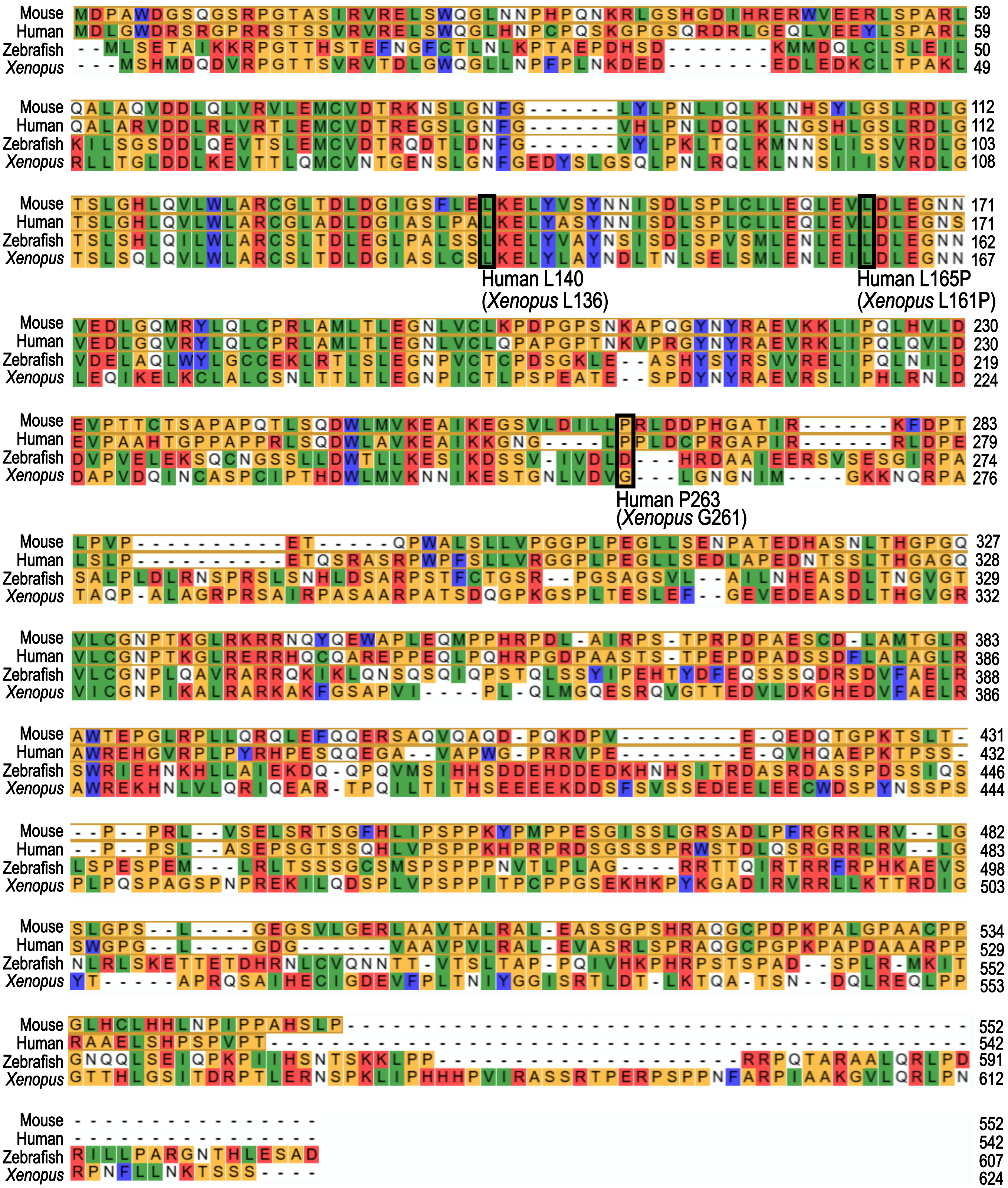

LRRC56 clustal analysis on mouse (Q8K375), human (Q8IYG6), zebrafish (A0A8M3APS4) and *Xenopus* laevis (A0A8J0V4N4). (UniProt ID). P263* allele results in a truncation of the LRRC56 IDR c-term region, *Xenopus* G261* is equivalent to this deletion.

Reyes-Nava et al 2025, Supplemental Figure 1

bioRxiv preprint doi: https://doi.org/10.1101/2025.07.02.662827; this version posted July 5, 2025. The copyright holder for this preprint (which was not certified by peer review) is the author/funder, who has granted bioRxiv a license to display the preprint in perpetuity. It is made available under aCC-BY 4.0 International license.

Supplemental Figure 1. **Alignment of vertebrate Lrrc56 proteins**. Multiple sequence alignment showing conserved Lrrc56 residues across mouse, human, zebrafish, and *Xenopus.* Rectangles indicate conserved ciliopathy loci characterized here.

bioRxiv preprint doi: https://doi.org/10.1101/2025.07.02.662827; this version posted July 5, 2025. The copyright holder for this preprint (which was not certified by peer review) is the author/funder, who has granted bioRxiv a license to display the preprint in perpetuity. It is made available under aCC-BY 4.0 International license.

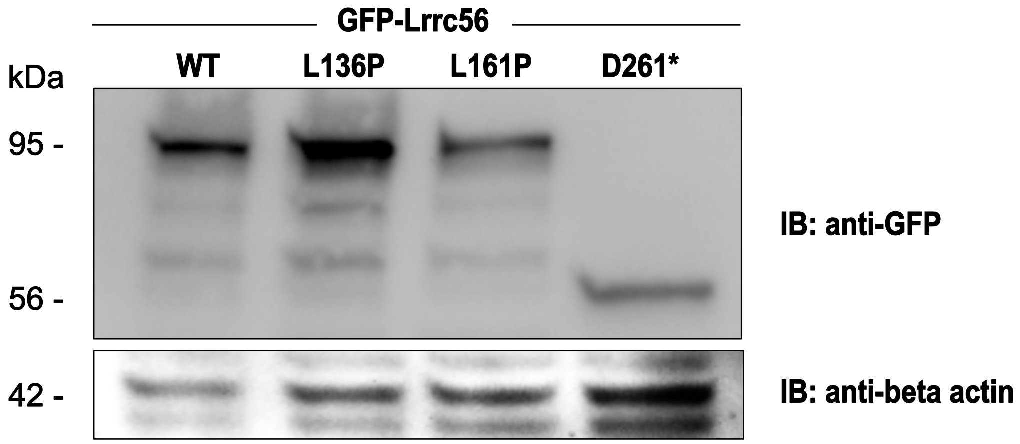

bioRxiv preprint doi: https://doi.org/10.1101/2025.07.02.662827; this version posted July 5, 2025. The copyright holder for this preprint (which was not certified by peer review) is the author/funder, who has granted bioRxiv a license to display the preprint in perpetuity. It is made available under aCC-BY 4.0 International license.

Supplemental Figure 2. **Lrrc56 ciliopathy variants protein abundance.**

Western blot showing protein levels for indicated disease alleles when expressed in *Xenopus* embryos. *Xenopus* variants (human): L136P (L140P), L161P (L165P), G261* (P263*). Western blot of total protein from N=20 embryos NF 25, injected with 80pg of GFP-Lrrc56 WT, L136P, L161P and 160pg of D261*. Anti GFP 1:200 and Anti B-actin housekeeping control (1:10,000).

bioRxiv preprint doi: https://doi.org/10.1101/2025.07.02.662827; this version posted July 5, 2025. The copyright holder for this preprint (which was not certified by peer review) is the author/funder, who has granted bioRxiv a license to display the preprint in perpetuity. It is made available under aCC-BY 4.0 International license.

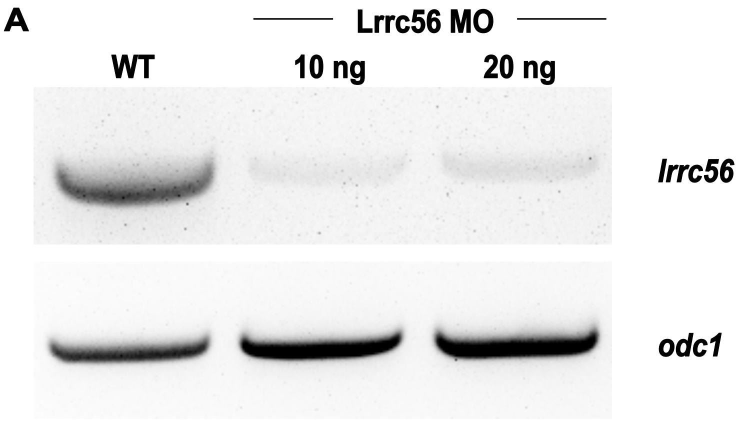

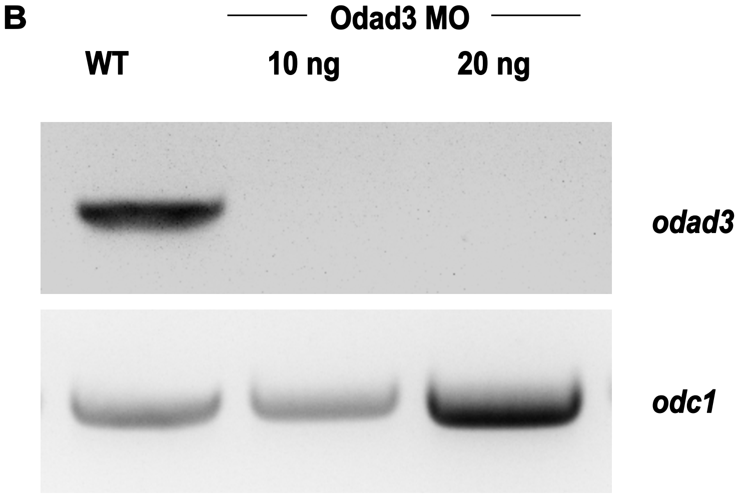

bioRxiv preprint doi: https://doi.org/10.1101/2025.07.02.662827; this version posted July 5, 2025. The copyright holder for this preprint (which was not certified by peer review) is the author/funder, who has granted bioRxiv a license to display the preprint in perpetuity. It is made available under aCC-BY 4.0 International license.

Supplemental Figure 3. Validation of Lrrc56 and Odad3 splice-blocking morpholino efficiency by RT-PCR. (A) Gel image of RT-PCR of *lrrc56* and *odc1* mRNA levels in wildtype control (WT), Lrrc56 MO 10ng and 20ng injected embryos. (B) Gel image of RT-PCR of *odad3* and *odc1* mRNA levels in wildtype control (WT), Lrrc56 MO 10ng and 20ng injected embryos.

bioRxiv preprint doi: https://doi.org/10.1101/2025.07.02.662827; this version posted July 5, 2025. The copyright holder for this preprint (which was not certified by peer review) is the author/funder, who has granted bioRxiv a license to display the preprint in perpetuity. It is made available under aCC-BY 4.0 International license.

AP-MS workflow

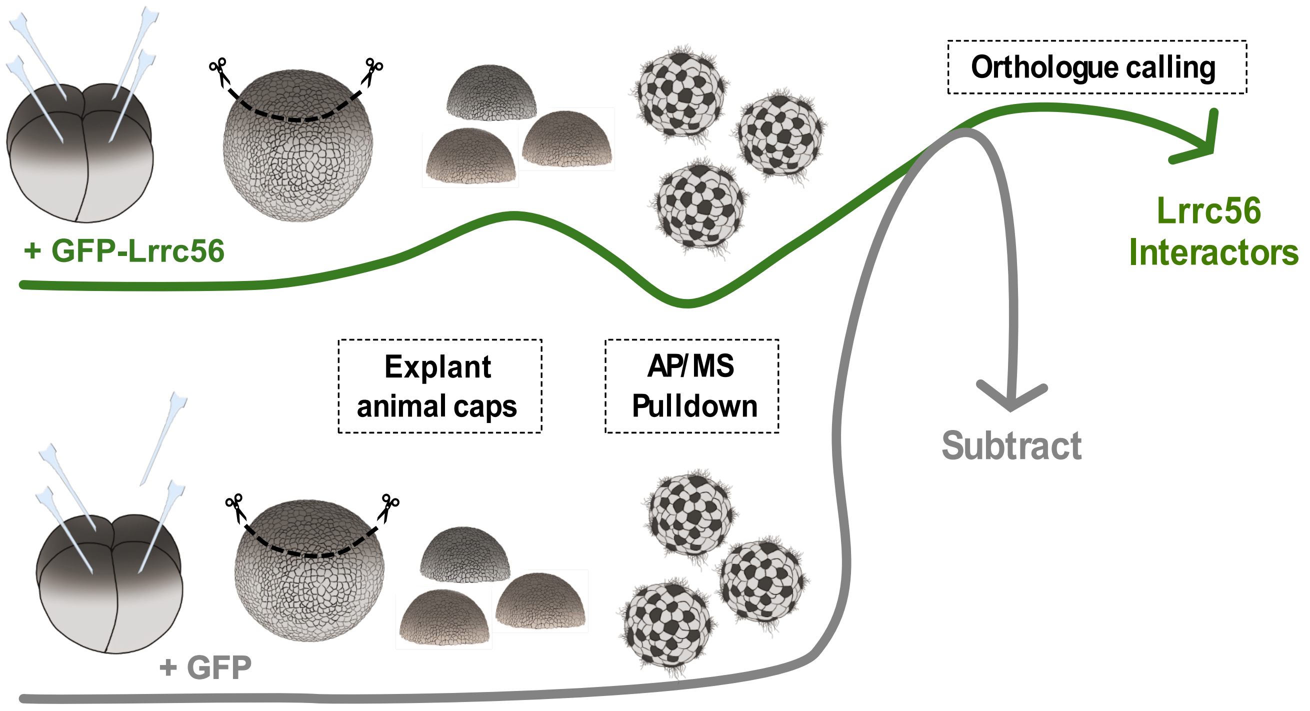

bioRxiv preprint doi: https://doi.org/10.1101/2025.07.02.662827; this version posted July 5, 2025. The copyright holder for this preprint (which was not certified by peer review) is the author/funder, who has granted bioRxiv a license to display the preprint in perpetuity. It is made available under aCC-BY 4.0 International license.

Supplemental Figure 4. Schematic of AP-MS workflow for identification of *in vivo* Lrrc56 interactors. A plasmid encoding GFP-tagged Lrrc56 under the control of the MCC-specific α-tubulin promoter was injected into Xenopus embryos at the 2–4 cell stage (stage 3). Animal cap explants were dissected at stage 8 and cultured until the early stage of ciliogenesis (stage 23). Explants were then harvested and subjected to GFP-based immunoprecipitation followed by affinity purification mass spectrometry (AP-MS). A parallel experiment using unfused GFP was performed to account for non-specific interactions, and these were subtracted from the experimental dataset to identify specific Lrrc56 interactors.

bioRxiv preprint doi: https://doi.org/10.1101/2025.07.02.662827; this version posted July 5, 2025. The copyright holder for this preprint (which was not certified by peer review) is the author/funder, who has granted bioRxiv a license to display the preprint in perpetuity. It is made available under aCC-BY 4.0 International license.

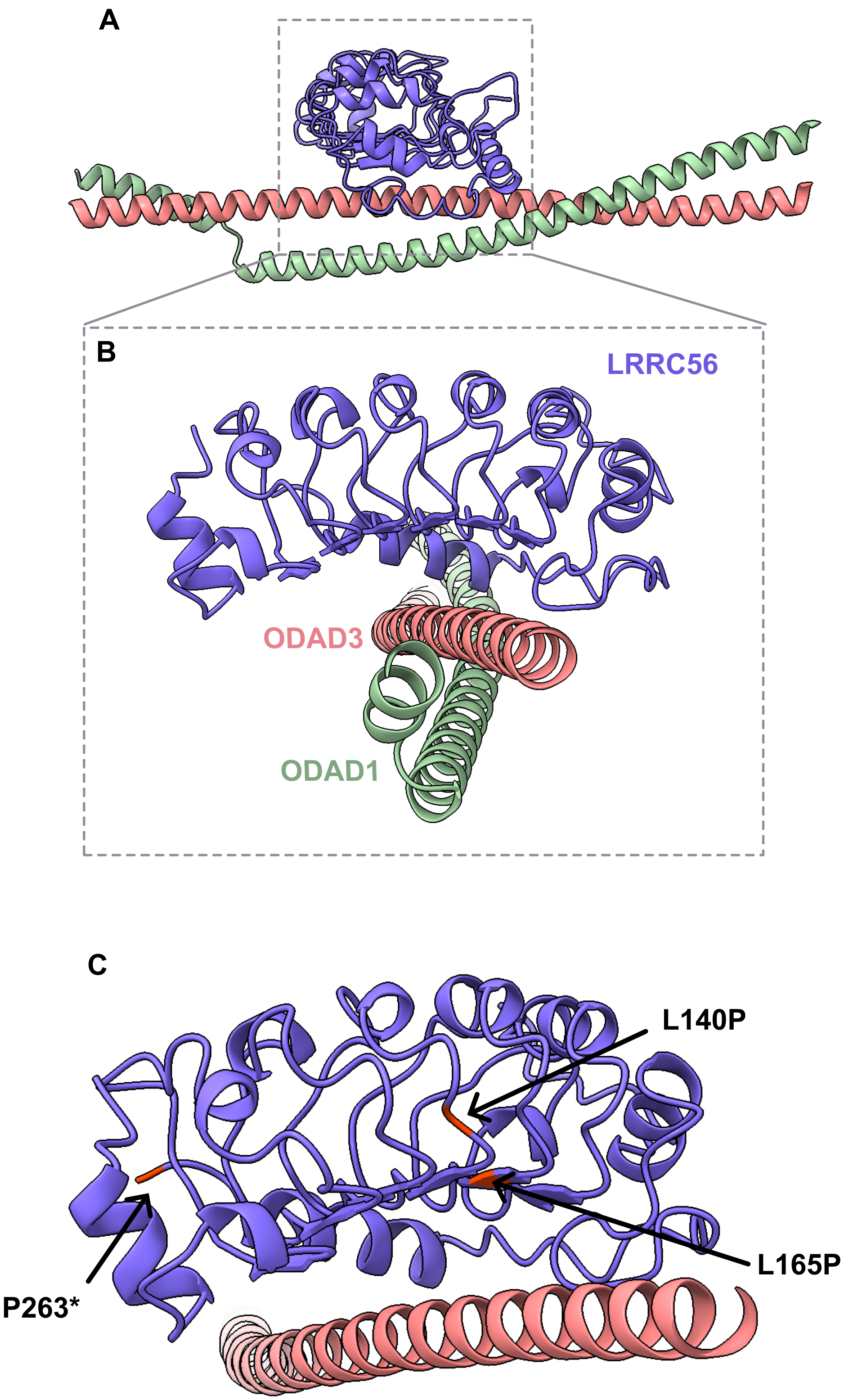

bioRxiv preprint doi: https://doi.org/10.1101/2025.07.02.662827; this version posted July 5, 2025. The copyright holder for this preprint (which was not certified by peer review) is the author/funder, who has granted bioRxiv a license to display the preprint in perpetuity. It is made available under aCC-BY 4.0 International license.

Supplemental Figure 5. Human AF3 model of LRRC56, ODAD3 and ODAD1.

(A) AlphaFold3-predicted structure of human LRRC56 and its interactors, ODAD3 and ODAD1. Each monomer is color-coded as indicated. The model includes residues 53-263 of LRRC56, 159-315 of ODAD3, and 80-224 of ODAD1.

(B) Enlarged view of the LRRC56–ODAD3 interface

(C) Close-up of the LRRC56–ODAD3 interface in the AlphaFold3 model, highlighting the positions of Lrrc56 ciliopathy-associated variants (L140P, L165P, and P263*).
